# Robotic right hemicolectomy with intracorporeal anastomosis versus laparoscopic right hemicolectomy with extracorporeal anastomosis (PRORHEM): protocol for a randomized controlled trial

**DOI:** 10.1186/s12893-026-03990-1

**Published:** 2026-07-08

**Authors:** Jeremy Meyer, Emilie Liot, Guillaume Meurette, Christian Toso, Frédéric Ris

**Affiliations:** 1 Division of Digestive Surgery, University Hospitals of Geneva, Rue Gabrielle-Perret-Gentil 4, Genève 14, 1211 Switzerland; 2 Medical School, University of Geneva, Rue Michel Servet 1, Geneva, 1205 Switzerland

**Keywords:** Robotic surgery, Colorectal surgery, Right hemicolectomy, Colorectal cancer, Incisional hernia.

## Abstract

**Background:**

In laparoscopic right hemicolectomy (lapRHC) for colorectal cancer, intra-corporeal anastomosis (ICA) has been associated with faster recovery and lower incidence of incisional hernia (IH) compared with extra-corporeal anastomosis (ECA). However, due to technical constraints, ICA is not widely adopted, and ECA remains the standard approach. Robotic surgery, with enhanced dexterity and suturing capability, facilitates ICA and may therefore improve short-term outcomes and reduce IH incidence.

**Methods:**

We designed a monocentric, double-blind randomized controlled trial to compare robotic right hemicolectomy (robRHC) with D2 lymphadenectomy, ICA, and off-midline extraction (fully minimally invasive) versus lapRHC with D2 lymphadenectomy, ECA, and midline extraction (standard of care). The primary endpoint is bowel recovery, assessed by time to first passage of stool. Secondary endpoints include perioperative safety (morbidity, mortality, resection margins, lymph node yield), length of stay, IH incidence, and patient-reported esthetic and quality-of-life outcomes. Based on a hypothesized 1-day reduction in bowel recovery, a total of 70 patients will be randomized.

**Ethics and dissemination:**

The trial complies with the Declaration of Helsinki, ICH-GCP, and Swiss regulatory standards. It has been approved by the local ethics committee (CCER 2024 − 00975) and registered in ClinicalTrials.gov (NCT06067620, 09.05.2024).

**Supplementary Information:**

The online version contains supplementary material available at https://doi.org/10.1186/s12893-026-03990-1.

## Introduction

Right hemicolectomy accounts for nearly one-third of colorectal cancer resections [1]. Laparoscopic right hemicolectomy is currently the standard treatment for localized right-sided colon adenocarcinoma [2–6]. During laparoscopic right hemicolectomy, intra-corporeal anastomosis offers several advantages over extra-corporeal anastomosis, including shorter hospital stay [7–17], reduced postoperative pain, faster bowel recovery, lower risk of surgical site infection, and smaller incisions. Intra-corporeal anastomosis also permits off-midline specimen extraction [9, 16], which is associated with a lower incidence of incisional hernia compared with midline extraction used in extra-corporeal anastomosis [18–21].

Although intra-corporeal anastomosis can technically be performed laparoscopically, it remains less frequently adopted in routine practice due to technical complexity and ergonomic constraints. Large observational studies indicate that extra-corporeal anastomosis remains the predominant approach during laparoscopic right hemicolectomy [22–25]. Robotic platforms facilitate intracorporeal suturing and therefore allow surgeons to perform a fully minimally invasive procedure combining intra-corporeal anastomosis with off-midline specimen extraction [23, 26, 27]. Observational studies further suggest that robotic right hemicolectomy reduces blood loss [28–30], complications [29, 30], conversion rates [28], and length of stay [28], while improving bowel recovery [29, 30], compared with laparoscopic right hemicolectomy. Nevertheless, robotic right hemicolectomy remains uncommon, performed in fewer than 10% of minimally invasive right hemicolectomies in Denmark [23] and under 5% in the USA [31]. Thus, laparoscopic right hemicolectomy with extracorporeal anastomosis and midline extraction remains the standard minimally invasive approach [22–25].

We hypothesize that robotic right hemicolectomy enables a fully minimally invasive surgical strategy by facilitating intracorporeal anastomosis and off-midline specimen extraction. Although intracorporeal anastomosis can technically be performed laparoscopically, it remains less frequently adopted in routine practice due to ergonomic limitations and technical complexity. Consequently, laparoscopic right hemicolectomy with extracorporeal anastomosis and midline extraction remains the predominant minimally invasive approach. The present trial therefore compares two surgical strategies as they are implemented in contemporary practice, rather than isolating a single technical variable. This pragmatic approach reflects the actual clinical decision faced by surgeons and institutions considering robotic adoption, and maximizes external validity.

The primary aim of this randomized trial is to determine whether robotic right hemicolectomy with intra-corporeal anastomosis reduces time to first passage of stool by one day compared with laparoscopic right hemicolectomy with extra-corporeal anastomosis. The secondary objective is to assess whether robotic right hemicolectomy with intra-corporeal anastomosis lowers the one-year incidence of incisional hernia.

## Methods and analysis

This study protocol was developed and is reported in accordance with the Standard Protocol Items: Recommendations for Interventional Trials (SPIRIT) guidelines [32] (Table S1).

### Study design

The PRORHEM trial is a prospective, single-center, double-blind, randomized controlled trial comparing robotic right hemicolectomy with intra-corporeal anastomosis against laparoscopic right hemicolectomy with extra-corporeal anastomosis. The study is conducted at the Digestive Surgery Division of the University Hospitals of Geneva and adheres to the Declaration of Helsinki, ICH-GCP guidelines, and Swiss regulatory requirements (ClinO, HRA). Approval was obtained from the Geneva Cantonal Ethics Committee (CCER 2024 − 00975), and the study is registered at ClinicalTrials.gov (NCT06067620, registered 09.05.2024).

### Study setting

All procedures are performed by three senior colorectal surgeons at our institution. Each surgeon is board-certified in general surgery, digestive surgery, and coloproctology and practices in an ECC-accredited colorectal cancer center. All surgeons have extensive experience in both laparoscopic and robotic colorectal surgery and have surpassed the reported learning curves for minimally invasive right hemicolectomy. Surgical procedures are distributed according to routine operating schedules to minimize operator-related bias.

### Participants

#### Eligibility criteria

##### Inclusion criteria

Adults (≥ 18 years) undergoing elective minimally invasive right hemicolectomy for non-metastatic adenocarcinoma staged cT1–T3, N0–2, M0 located in the appendix, caecum, ascending colon, or hepatic flexure. Curative intent must be planned.

##### Exclusion Criteria

Patients will be excluded for any of the following:

- Age < 18 years.
- Refusal of surgery, planned open approach, or high risk of conversion to open surgery ; history of midline laparotomy.
- Emergency surgery.
- Prior colorectal resection (except appendectomy).
- Hereditary colorectal cancer or inflammatory bowel disease.
- Extended transverse colectomy (left branch of middle colic artery).
- Combined or synchronous colorectal or multivisceral resections.
- Locally advanced (cT4) or metastatic (cM+) disease.
- Neoadjuvant treatment.
- Pregnancy or lactation.
- Immunosuppression.
- No plan for ileocolic anastomosis.
- Inability to provide written informed consent.

#### Recruitment and consent

Eligible patients are identified during routine preoperative outpatient visits. A consultant colorectal surgeon provides written and oral information regarding trial objectives, procedures, and risks. Patients must sign the informed consent form at least 48 h prior to randomization. Withdrawal is allowed at any time without impact on care. Patients withdrawing before randomization are not included in analyses.

## Randomization and allocation concealment

Randomization is performed using the REDCap randomization module with a 1:1 allocation ratio, stratified by operating-theatre availability to avoid imbalances caused by robotic platform scheduling.

Allocation concealment is ensured automatically by REDCap. Only the operating theatre scheduling team is informed of the assigned modality. Surgeons cannot be blinded for practical reasons.

### Blinding

Blinding applies to patients and all outcome assessors until discharge.

To conceal incisions, standardized abdominal dressings are applied postoperatively, covering all potential port sites from both techniques, including unused sites. During hospitalization, the electronic operative note is temporarily masked and records only “Minimally invasive right hemicolectomy – PRORHEM trial.”

Dressings are removed, and the final operative note is released only after discharge. As a result, all measurements of the primary outcome occur under fully blinded conditions. Blinding cannot be maintained for long-term outcomes (e.g., incisional hernia, esthetic evaluation).

Any unblinding required for clinical reasons is recorded and the patient remains in intention-to-treat analysis.

### Interventions

All procedures are performed by a team of four EBSQ-certified colorectal surgeons with extensive experience in both approaches and who have surpassed the learning curve thresholds. All surgeons adhere strictly to standardized technical steps defined in the protocol.

All patients follow an identical enhanced recovery after surgery (ERAS) pathway, including oral tobramycin/colistin for 2 days preoperatively, carbohydrate loading (unless diabetic), and no mechanical bowel preparation.

#### Robotic right hemicolectomy with intracorporeal anastomosis (Experimental Arm)

Robotic right hemicolectomy is performed using the da Vinci 5 platform, following a standardized and previously published technique developed at our institution.

##### Patient positioning and access

Patients are placed in a modified Lloyd-Davies position with slight Trendelenburg and left tilt, ensuring flat thighs to avoid robotic arm conflict. Pneumoperitoneum is induced using a Veress needle, and the camera is inserted through a 12-mm AirSeal port in the left flank.

##### Port placement and docking

Three 8-mm robotic ports and one 12-mm stapling port are inserted along a straight line from the planned Pfannenstiel extraction site toward the left subcostal margin. The Xi robotic column is docked from the patient’s right side. Instruments are standardized to ensure reproducibility: fenestrated bipolar forceps (port 1), 30° camera (port 2), monopolar scissors (port 3), and small grasping retractor (port 4).

##### Bottom-up dissection

A bottom-up medial approach is performed. The peritoneum is incised along the mesenteric root; the embryological plane is developed toward the duodenum, exposing the superior mesenteric vessels. The duodenum and pancreatic head are mobilized and protected with a laparoscopic swab.

##### Lymphadenectomy and vascular ligation

The ileocolic pedicle is skeletonized and controlled at its origin using Hem-o-lock clips or the robotic vessel sealer. The right branch of the middle colic artery is similarly treated.

##### Mobilization

The hepatic flexure, ascending colon, and terminal ileum are mobilized via combined medial and lateral release, with restoration of anatomical orientation before transection.

##### Assessment of perfusion and bowel transection

Using the Firefly system, indocyanine green fluorescence is performed before transection. The terminal ileum and transverse colon are divided using the SureForm robotic stapler.

##### Intracorporeal anastomosis (ICA)

A standardized isoperistaltic side-to-side stapled ileocolic anastomosis is created intracorporeally. The enterotomy is closed with a continuous 15-cm barbed suture (V-Loc™ 3 − 0), following a unidirectional running technique ensuring full-thickness closure.

##### Specimen extraction and closure

The specimen is placed in a protection bag and extracted through a Pfannenstiel incision, ensuring an off-midline extraction site. Fascial closure follows standard institutional techniques using slowly absorbable running sutures (Maxon 2 − 0). All skin incisions are closed with Monocryl 3 − 0 and skin glue. Standardized wound dressings covering all potential port sites are applied to maintain blinding.

#### Laparoscopic right hemicolectomy with extracorporeal anastomosis (Control Arm)

The laparoscopic technique follows a standardized multiport bottom-up approach, largely paralleling the robotic steps but preceding extracorporeal extraction.

##### Patient positioning and access

Open laparoscopy is performed at the umbilicus. Pneumoperitoneum is established and three additional 5-mm ports are placed: left iliac fossa, left flank midclavicular, and suprapubic.

##### Bottom-up dissection

A bottom-up medial approach is performed. The peritoneum is incised along the mesenteric root; the embryological plane is developed toward the duodenum, exposing the superior mesenteric vessels. The duodenum and pancreatic head are mobilized and protected with a laparoscopic swab.

##### Lymphadenectomy and vascular ligation

The ileocolic pedicle is skeletonized and controlled at its origin using Hem-o-lock clips or the robotic vessel sealer. The right branch of the middle colic artery is similarly treated.

##### Mobilization

The hepatic flexure, ascending colon, and terminal ileum are mobilized via combined medial and lateral release, with restoration of anatomical orientation before transection.

##### Assessment of perfusion and bowel transection

Using the Arthrex system, indocyanine green fluorescence is performed before transection.

##### Extra-corporeal anastomosis

A supra-umbilical or periumbilical midline incision < 10 cm is created, protected by an Alexis retractor. Mesenteric division is performed extracorporeally. The terminal ileum and transverse colon are divided using linear staplers. A conventional side-to-side isoperistaltic ileocolic anastomosis is constructed.

##### Closure

Extraction site fascia is closed using running slowly absorbable sutures (Maxon 2 − 0). All skin incisions are closed with Monocryl 3 − 0 and skin glue. Standardized wound dressings covering all potential port sites are applied to maintain blinding.

### Outcomes measures

#### Primary outcome

Time to first passage of stool (hours), self-reported by patients using standardized forms and verified by a blinded research nurse. Time is measured from skin closure.

This endpoint was selected for four reasons. First, it was used as the primary outcome in the two most directly comparable randomized trials in this field [12, 33] enabling direct comparison of results. Second, it directly reflects the degree of bowel manipulation during surgery, which is the central mechanistic hypothesis of this trial. Third, unlike length of stay, it is not confounded by institutional discharge policies, social circumstances, or healthcare organization. Fourth, in our institution, return of bowel function is a discharge criterion within the ERAS protocol, meaning a 1-day reduction has direct implications for hospitalization duration and patient experience. The 1-day minimal clinically important difference used for sample size calculation is conservative, given that the observed difference between intracorporeal and extracorporeal anastomosis in prior randomized trials exceeds 2 days. We acknowledge that this endpoint may not translate directly into shorter hospitalization in all healthcare systems, and this limitation is discussed below.

#### Secondary outcomes

##### Operative outcomes

- Duration of surgery.
- Blood loss and transfusion.
- Intraoperative complications.
- Conversion to open surgery (defined as any unplanned extraction site exceeding 10 cm, or any unplanned switch to open surgery not anticipated at the time of randomization; planned Pfannenstiel and midline extractions within the protocol are not considered conversions).
- Extraction-site length.
- Stoma creation.

##### Postoperative outcomes

- CRP, WBC, and Hb at post-operative days 1–5.
- Time to first flatus.
- Postoperative ileus (defined as absence of stool passage and intolerance to oral feeding beyond postoperative day 3 in the absence of mechanical obstruction).
- Length of stay.
- 30-day postoperative complications graded according to the Clavien–Dindo classification.
- 30-day reoperation.
- 30-day mortality.

##### Surgical quality

- Proximal and distal margins.
- Lymph node yield.
- Histopathology and TNM stage.

##### Patient-reported outcomes

- GIQLI score at 30 days and 1 year.
- QoR-15 score at POH12-POD7.
- Esthetic numeric analogue scale (ANA) at 30 days and 1 year.

##### Long-term outcomes

- 1-year incidence of incisional hernia (clinical + CT).

Assessment time points are summarized in (Table 1).

**Table 1:** Schedule of enrollment, interventions and assessments

	TRIAL PERIOD												
	Enrollment		Post-randomization										Close-out
TIMEPOINT	-t_i_ to 0	0	POH12	POD1	POD2	POD3	POD4	POD5	POD6	POD7	Month 1	Year 1	
ENROLLMENT:													
Eligibility screen	x												
Informed consent	x												
Randomization	x												
INTERVENTION/ COMPARATOR:													
Robotic right hemicolectom^c^		x											
Laparoscopic right hemicolectomy		x											
ASSESSMENTS:													
First flatus			x	x	x	x	x	x	x	x			
First faeces			x	x	x	x	x	x	x	x			
Blood tests				x	x	x	x	x					
QoR-15 score			x	x	x	x	x	x	x	x			
Wound examination										x	x		
GIQLI score											x	x	
ANA score											x	x	
Extraction site measurement											x		
Extraction site CT + clinical examination												x	

### Sample size calculation

Based on Allaix et al. [12], the mean ± SD time to first stool after lapRHC with ECA is 4 ± 1.4 days. We hypothesize that robRHC with ICA reduces this by ≥ 1 day, a clinically relevant difference supported by prior reports. Using α = 0.05 (two-sided) and 80% power, 31 patients per arm are required. Allowing for 10% drop-out, we will enroll 70 patients (35 per arm). Statistical supervision is provided by our methodological unit.

### Statistical analysis

Analyses will be conducted on an intention-to-treat basis, including all randomized patients. A modified intention-to-treat analysis will exclude patients who were randomized but did not undergo the allocated procedure. A per-protocol analysis will also be performed as a sensitivity check.

Continuous variables (e.g. time to first stool, length of stay) will be summarized as means ± SD or medians with interquartile ranges, as appropriate. Between-group comparisons will use Student’s t-test or Mann–Whitney U test for continuous outcomes, and χ² or Fisher’s exact test for categorical outcomes. Time-to-event data will be analyzed using Kaplan–Meier curves and log-rank tests. Multivariable linear and logistic regression models will be applied to adjust for potential confounders.

All tests will be two-sided with α = 0.05. Secondary endpoints are considered exploratory and hypothesis-generating. Results will be reported with point estimates and 95% confidence intervals and interpreted with appropriate caution, without correction for multiplicity. Missing data for the primary endpoint will be carefully examined. If missingness exceeds predefined thresholds, multiple imputation may be considered according to standard statistical recommendations. Sensitivity analyses will be performed to assess the potential impact of missing data. Statistical analyses will be performed with Stata/SE 18 (StataCorp, College Station, TX, USA).

A detailed statistical analysis plan was developed in collaboration with a senior biostatistician from the institutional methodological unit prior to data collection and will be finalized before database lock and before access to outcome data.

### Safety monitoring

Robotic right hemicolectomy is a well-established procedure routinely performed at our institution as part of standard clinical care. For this reason, an independent data safety monitoring committee was not considered necessary for this investigator-initiated trial. However, patient safety will be closely monitored throughout the study.

### Data management

Data are recorded in REDCap and coded using unique identifiers. Paper CRFs and electronic backups are stored securely for 20 years, as per Swiss regulations.

## Discussion

This randomized controlled trial is designed to provide high-quality evidence on whether a fully minimally invasive robotic right hemicolectomy with intra-corporeal anastomosis offers superior postoperative recovery compared with the current real-world standard of laparoscopic right hemicolectomy with extra-corporeal anastomosis. Although observational studies have repeatedly suggested advantages of robotics—particularly regarding anastomotic technique, extraction-site location, postoperative recovery, and risk of incisional hernia—robust randomized data are lacking. Existing trials have not standardized the anastomotic method within treatment arms, limiting the ability to isolate the potential benefits of a fully minimally invasive approach. By mandating intra-corporeal anastomosis and off-midline extraction in the robotic arm, and extra-corporeal anastomosis with midline extraction in the laparoscopic arm, our trial evaluates the two techniques as they are actually practiced in most institutions, thereby maximizing clinical relevance.

Several methodological strengths reinforce the validity of this study. First, both patients and outcome assessors remain blinded until discharge through the combined use of standardized abdominal dressings and a temporary neutral operative note, ensuring unbiased measurement of the primary endpoint. Second, all procedures are performed by four EBSQ-certified colorectal surgeons, each beyond the learning curve for both techniques, and all committed to applying the standardized operative steps outlined in the protocol. This high level of expertise and consistency minimizes performance bias—a frequent limitation in surgical trials. Third, perioperative care is fully standardized through a single ERAS pathway, reducing variation in recovery attributable to non-surgical factors. Finally, the inclusion of multidimensional outcomes—functional recovery, postoperative morbidity, oncologic quality, patient-reported outcomes, and CT-based assessment of incisional hernia—allows a comprehensive evaluation of both short- and longer-term benefits.

However, this study also has limitations. The single-center design may restrict generalizability, particularly to lower-volume units or teams earlier in their learning curve. This trial was designed to evaluate composite surgical strategies as they are practiced in the real world, which maximizes clinical relevance but precludes causal attribution to any single component. The inability to isolate the individual contributions of the robotic platform, intracorporeal anastomosis, and off-midline extraction is an intentional feature of the pragmatic design rather than a methodological flaw. Future mechanistic trials isolating individual variables would be complementary to the present work. Blinding cannot be maintained beyond discharge, and therefore longer-term outcomes—including esthetic satisfaction and incisional hernia—are unblinded by necessity. Finally, the study is powered for the primary endpoint only and may not detect differences in rare events such as anastomotic leak or mortality. Additionally, while time to first passage of stool is a validated and physiologically meaningful measure of bowel recovery, it may not translate into shorter hospitalization in healthcare systems where discharge is not contingent on bowel function. However, length of stay is included as a key secondary outcome and will be fully reported.

Despite these limitations, this trial is expected to provide the most rigorous evidence to date comparing a fully standardized robotic minimally invasive approach with the laparoscopic standard of care. Given the increasing adoption of robotic platforms worldwide and the potential for clinically meaningful improvements in recovery and hernia prevention, these results may have important implications for surgical practice and healthcare resource utilization.

### Patient and public involvement

No patient and/or member of the public was involved in the conception of the trial.

## Trial status

The trial was prospectively registered on ClinicalTrials.gov (NCT06067620) on 09.05.2024, prior to enrolment of the first participant. Recruitment is planned to begin on 1 January 2026, with anticipated completion on 31 December 2028. At our institution, more than 40 right hemicolectomies for primary colon cancer are performed annually. At the time of submission of this manuscript, patient recruitment has not yet started.

## Supplementary Information

Supplementary Material 1.

## Data Availability

No datasets were generated or analysed for this study protocol. De-identified data generated during the trial will be available from the corresponding author upon reasonable request after publication of the trial results.
